# Genotype distribution of cervical HPV among Caribbean women in a population-based study in Martinique: The DEPIPAPUFR study

**DOI:** 10.1371/journal.pone.0257915

**Published:** 2021-10-07

**Authors:** Fatiha Najioullah, Marie-José Dorival, Clarisse Joachim, Christiane Dispagne, Jonathan Macni, Sylvie Abel, Caroline Sulpicy, Huguette Charpentier-Baltide, Danièle Sainte-Rose, Béatrice Salomon-Frechou, Moustapha Dieye, Jacqueline Véronique-Baudin, Maurice Pré, Maurice Marquet, Gaëlle Wan-Ajouhu, Eustase Janky, Didier Riethmuller, Raymond Cesaire, the DEPIPAPUFR study group

**Affiliations:** 1 CHU de Martinique, Pôle de Biologie - Pathologie, Laboratoire de Virologie, Martinique, France; 2 Laboratoire de Pathologie SERAL, Fort-de-France, Martinique, France; 3 CHU de Martinique, Pôle de Cancérologie Hématologie Urologie, UF1441 Registre Général des cancers de la Martinique, Martinique, France; 4 Residence de Cluny, Martinique, France; 5 Service de Maladies Infectieuses et Tropicales, CHU de Martinique, Martinique, France; 6 Rue Victor Schoelcher, Martinique, France; 7 CHU de Martinique, Martinique, France; 8 Avenue François Mitterand, Martinique, France; 9 Centre Hospitalier Angoulême, Angoulême, France; 10 All Lauriers, Le Robert, Martinique, France; 11 Route de Redoute, Martinique, France; 12 CHU de Guadeloupe-Laboratory CELTEC Cancer and Environment EA4546, Université des Antilles, Guadeloupe, France; 13 Service de Gynécologie Obstétrique, CHRU de Besançon - Hôpital Jean Minjoz, Besançon, France; Penn State University School of Medicine, UNITED STATES

## Abstract

The Caribbean ranks seventh among the world regions most affected by cervical cancer. HPV-prevalence and genotype distributions also differ from regions. Knowledge of HPV genotype profiles is important for patients care and HPV vaccination implementation. The objective of this study was to describe HPV genotype distribution and risk factors in a population-based cohort of women in Martinique. In this study, 1312 women were included and underwent cervical cancer screening with successful sample collection between 2009 and 2014. Sociodemographic and clinical variables were recorded. Cytological examination of cervical vaginal smear was performed and classified(Bethesda). Detection of HPV DNA was performed with the PapilloCheck^©^ Kit from Greiner Bio-one. Genotypes were analyzed for18 high-risk HPV (hrHPV) and 6low-risk HPV(lrHPV) types. A total of 1075 women were included with a mean age of 49.1±10.5 years. HPV prevalence was 27.6% (297/1075) with 19.4% (209/1075) women with only hrHPV, 5.3% (57/1075) with only lrHPV. Multiple infections (hrHPV/lrHPV) were detected in 31/240 cases of hrHPV (12.9%). A total of 353 hrHPV genotypes were analyzed; the most common HPV types were HPV51 (11.0%), HPV68 (10.8%), HPV53 (9.1%) and HPV 52 (7.1%). HPV16 and HPV18 represented respectively 4.8% and 4.0% of hrHPV genotypes. Abnormal cytology was observed in 34 cases (3.2%), with 14 ASCUS (1.3%), 10 LSIL (0.9%), 5 HSIL (0.5%), 3 ASC-H (0.3%) and 2 AGC (0.2%). Fifteen (44.1%) were hrHPV and 4 (14.7%) lrHPV; 7 cases of hrPHV were in the age-group 25–34 years. Among 1041cases of normal cytology, 225 had positive hrHPV detection (21.6%). This is the first population-based study of HPV profiles in our country, and we found a high prevalence of hrHPV. The most common genotypes were HPV51, 68, 53. These results could serve for cancer vaccination strategies and HPV surveillance in Martinique.

## Introduction

Cervical cancer is a global public health problem caused by persistent human papillomaviruses (HPV) infection leading to malignancy. In women, it ranks fourth in terms of cancer incidence and mortality worldwide, with respectively rates of 13.1 and 6.9 per 100 000 women. In the Caribbean, cervical cancer ranks in third position in terms of incidence and mortality (15.5/100 000 women and 8.5/100 000 women).

In France, cervical cancer is ranked twelfth in terms of incidence (6.7/100 000 women) and tenth (2.3/100 000) in terms of mortality [1]. In the Caribbean, three French overseas territories exist: Martinique, Guadeloupe and French Guiana; these regions have the same health national system of Metropolitan France. The number of incident cervical cancer cases per year is 28 in Guadeloupe, 26 in Martinique, and 25 in French Guiana. Compared to Metropolitan France, incidence is higher in Guadeloupe (8.7 per 100,000 person-years), and considerably higher in French Guiana (at 22.4), while in Martinique, incidence (7.2 cases per 100,000 person-years) is not significantly different than in mainland France (3 159 cases; 6.6 cases per 100,000 person-years) [2].

Martinique is a French department; in total, there were 380,877 inhabitants in Martinique as of 1 January 2015, and 3,057 deaths were recorded that year, with a rising trend since 1990. The mortality rate increased from 6.9 per thousand in 2000 to 8.1 per thousand in 2015, largely related to population aging [3]. In 2015, 806 cancer-related deaths were recorded, corresponding to 26.4% of all deaths that year. In the regions of Guadeloupe, Martinique and French Guiana, cervical cancer is diagnosed in an average of 79 women per year, and represents 5.6% of all incident cancers in women [4].

The natural history of cervical cancer develops over a period of 15 to 20 years, along a continuum of pre-cancerous histological lesions (intraepithelial cervical neoplasia, CIN) that result from persisting infection with high-oncogenic-risk serotypes of the human papillomavirus (HPV). HPV infection is generally transitory and the majority of low grade lesions associated with infection heal spontaneously and do not systematically progress towards the stage of pre-cancerous or invasive lesions. In France, cervical cancer screening primarily consists in regular and recommended (individual) screening by smear test among women aged 25 to 65 years on a three-yearly schedule, after 2 normal smear tests performed at one year’s interval. If the smear test result shows Atypical Squamous Cell of Unknowned Significance (ASC-US), it is recommended to search for high-risk HPV (hrHPV) types.

When considering cancer prevention strategies, knowledge of HPV genotype profiles is important for HPV vaccination implementation and screening of cervical cancer. There is a huge need to get access on HPV genotype distribution in Martinique, due to the higher incidence of cervical cancer in our region. To perform a monitoring of the HPV evolution in the next years, we need to first describe the epidemiological context in Martinique, to better improve the cervical screening and the HPV vaccination in the future. The objective of this study was thus to describe HPV genotype distribution in a cohort of women in Martinique. We also studied clearance of HPV infection among women who presented normal cervical cytology and performed follow-up to assess outcomes developed cancer in situ or invasive cervical cancer.

## Methods

### Population

We performed a prospective study among women aged 25 to 65 years who had complementary health insurance with a single company (Mutuelle UFR), were resident in Martinique, and underwent a smear test for cervical cancer screening. Women who were included had not received HPV vaccination. The exclusion criteria were a history of total hysterectomy, or a history of cervical lesions of grade CIN2 or higher. Our study was based on a sample of 14,333 women who were invited to participate to this study in Martinique.

Training and information sessions were organised for general practitioners, specialists and midwives regarding the use of liquid medium for combined screening. Women participating in the study could attend the physician of their choice to undergo screening, as long as the physician was participating in the study. Information leaflets provided details of cervical cancer screening, the study procedures, follow-up after the smear test and HPV test performed, and an informed consent document was also included, to receive a collection kit with the liquid medium vial.

### Screening strategy

The screening strategy comprised both a smear test and a HPV test. Invitations were sent out from July 2009 to February 2011 with reminders by the insurance company to all its eligible members. Women aged 25 to 65 were invited to participate in age groups, starting with women aged 50 to 65, then women aged 40 to 49, then 25 to 39 years. Women were included between 1stJuly 2009 and 28thFebruary 2014. The validated liquid media chosen for this study were NovaPrep (NovaCyt France) and Easyfix (Labonord).

Samples for cytological analysis of smear tests were prepared using the automated NOVACYT system. The interpretation of smear test results was done according to the 2014 Bethesda classification [5].

Samples were classified as atypical squamous cells of undetermined significance (ASC-US), low-grade squamous intraepithelial lesion (LSIL), or high-grade squamous intraepithelial lesion (HSIL), Atypical Squamous Cell evocating High grade lesion (ASC-H), Atypical Glandular Cells (AGC). In case of abnormal cytology, a histological sample was obtained by colposcopy-directed biopsy or endocervical curettage. Confirmation of pre-cancerous lesions of the cervix and invasive cancers was done by histological examination of biopsy samples. Pathology charts were analysed using the electronic database DIAMIC software.

Extraction of HPV DNA was performed using Easymag from Biomérieux. Detection of HPV DNA was performed with the PapilloCheck^©^ Kit by the Virology Laboratory of the University Hospital of Martinique. Genotypes were analyzed for high-risk HPV (hrHPV) and low-risk HPV (lrHPV). The PapilloCheck^®^ test allows the identification of DNA from eighteen hrHPV types, namely: 16, 18, 31, 33, 35, 39, 45, 51, 52, 53, 56, 58, 59, 66, 68, 70, 73, 82 and six lrHPV: types 6, 11, 40, 42, 43, 44/55. Genotypes were also classified according to the IARC Monograph Working Group in Alpha HPV types [6].

Appropriate management was provided for women in whom anomalies were found. French recommendations regarding the management of patients with abnormal smear test are those of the French national cancer institute (INCa) [7].

For positive HPV tests, a one-year follow-up was performed with both smear test and HPV test. If both tests were negative, a smear test was performed at 3 years. If the smear test was still negative with a positive HPV test, a one-year follow-up was performed once again. If both the smear and HPV tests were positive, a colposcopy was performed. If the colposcopy was negative, a one-year follow-up was performed. Follow-up of the women in the study made it possible to identify cases of cancer recorded in the database of the General Cancer Registry of Martinique. Cancer cases occurring between 2009 and 2016 were identified after data extraction.

### Statistical analysis

The prevalence rate of HPV infection in the cohort was calculated with 95% confidence intervals (CIs). Prevalence was obtained by dividing the number of women with normal cytology infected by at least one HPV genotype by the total number of women. Age is described using the mean, median and interquartile range (Q1–Q3). Stratification according to age was performed within each age group: 25–34, 35–44, 45–54, 55–64, and 65 years or more.

Sociodemographic and clinical variables were recorded, namely marital status, profession, number of children and age at first intercourse (<18year or ≥ 18year). Socio-professional categories were noted in four groups: group 1 (i.e. farmers, craftsmen, retailers, intermediate occupations); group 2 (i.e. labourers, lower-grade employees); group 3 (i.e. retired people) and group 4 (others, including never-worked or long-term unemployed, students). Analyses were performed using SAS version 9.4 (SAS Institute Inc., Cary, NC). Results are presented as mean ± standard deviation or number and percentage. Cross-tabulations were analyzed by Chi-square test or Fisher’s exact test as appropriate. Comparisons of prevalence rates with other countries were based on p-values. For all analyses, two-sided p-values of less than 0.05 were considered statistically significant.

### Ethics statement

The study was approved by the Ethics Committee (CPP Bordeaux) on 25 March 2009 under the number 2008-A01658-47. All women provided written informed consent.

## Results

**Fig 1:**
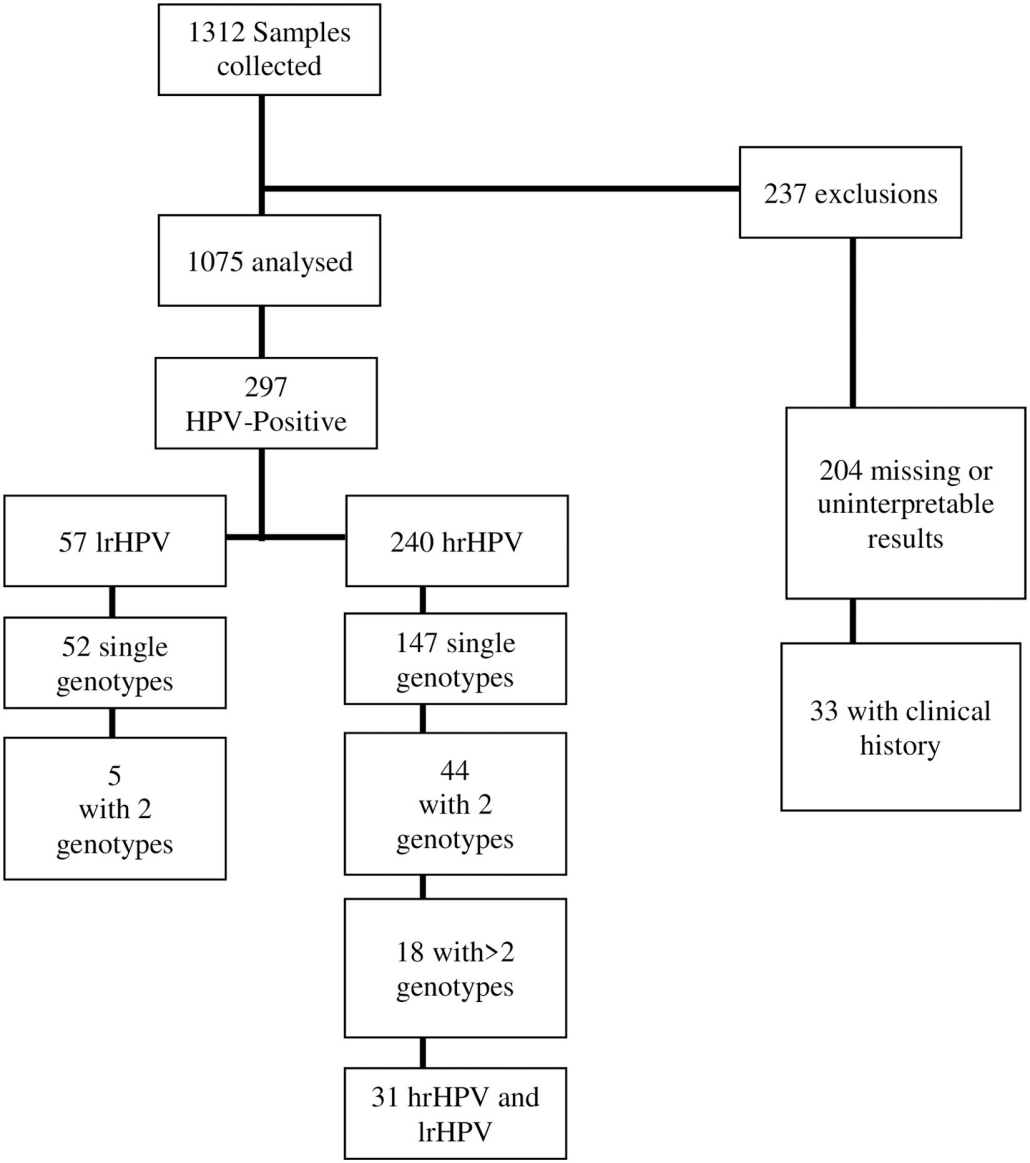
Flowchart of the study population and number of genotypes identified.

Overall, 1312 women were included and underwent cervical cancer screening with successful sample collection. A total of 237 women were excluded (204 had a missing smear or HPV test or uninterpretable results, and 33 had a clinical history according to the study methodology). Fig 1 shows a flow chart of the study population. A final total of 1075 women were included in the present analysis, with a mean age of 49.1±10.5 years.

HPV prevalence was 27.6% (297/1075) with a total of 240 (22.3%) women with at least one hrHPV genotype detected. A total of 19.4% (209/1075) of women had hrHPV, 5.3% (57/1075) having only lrHPV, and multiple infections (hrHPV/lrHPV) detected in 31/240 cases of hrHPV (12.9%). Among these, 164 had a single viral genotype (68.3%), 51 (21.2%) had 2 genotypes identified, and 25 (10.4%) had more than 2 types of hrHPV identified.

Socio-demographic characteristics of the study population are presented in Table 1.

**Table 1:** Socio-demographic characteristics of the study population according to HR-HPV status, N = 1075.

	All							HPV					
			HPV negative		HPV positive		*p*	lrHPV		hrHPV		lrHPV/hrHPV	
	n	%	n	%	n	%		n	%	n	%	n	%
	1075	100.0%	778	72.4%	297	27.6%		57	19.2	209	70.4	31	10.4
Median age [IQR], yrs							0.212						
25–34	113	10.5	71	9.1	42	14.1		7	12.3	33	15.8	2	6.5
35–44	240	22.3	177	22.7	63	21.2		16	28.1	43	20.6	4	12.9
45–54	336	31.3	247	31.8	89	30.0		17	29.8	59	28.2	13	41.9
55–64	369	34.3	271	34.8	98	33.0		16	28.1	71	34.0	11	35.5
65	17	1.6	12	1.6	5	1.7		1	1.7	3	1.4	1	3.2
Total													
Marital status							0.139						
Conjugal status	555	54.9	415	56.3	140	51.1		22	42.3	98	50.3	20	74.1
Single	456	45.1	322	43.7	134	48.9		30	57.7	97	49.7	7	25.9
Unknown	64	-	41	-	23	-		5	-	14	-	4	-
Number of children							0.006						
None	163	16.5	105	14.6	58	21.3		13	25.0	42	22.0	2	6.4
1	224	22.6	153	21.4	71	26.1		17	32.7	42	22.0	3	9.7
2	331	33.5	257	35.8	74	27.2		10	19.2	57	29.8	12	38.7
≥ 3	271	27.4	202	28.2	69	25.4		12	23.1	50	26.2	7	22.6
Unknown	86	-	61	-	25	-		5	-	18	-	7	22.6
Age at 1^st^ intercourse							0.966						
<18	455	53.5	330	53.5	125	53.6		13	22.8	47	22.5	4	12.9
≥ 18	395	46.5	287	46.5	108	46.4		23	40.4	83	39.7	19	61.3
Unknown	225	-	161	-	64	-		21	36.8	79	37.8	8	25.8
Socio-professional group							0.636						
Group 1	484	45.0	342	44.0	142	47.9		23	40.3	103	49.3	16	51.6
Group 2	386	35.9	284	36.5	102	34.3		23	40.4	69	33.0	10	32.3
Group 3	89	8.3	64	8.2	25	8.4		3	5.3	19	9.1	3	9.7
Group 4	116	10.8	88	11.3	28	9.4		8	14.0	18	8.6	2	6.4

A total of 449 HPV genotypes were analyzed and 353 were hrHPV. Table 2 shows the distribution of HPV genotypes according to age groups. For hrHPV, the most common hrHPV types were HPV51 (11.0%), HPV68 (10.8%) and HPV53 (9.1%) and HPV 52 (7.1%). HPV16 and HPV 18 represented respectively 4.8% and 4.0% of hrHPV genotypes.

**Table 2:** HPV genotype distribution, for hrHPV and lrHPV genotypes, N = 449 genotypes.

	All		25–34		35–44		45–54		55–64		65 +	
	n	%	n	%	n	%	n	%	n	%	n	%
Total HPV genotypes	449	100.0	67	14.9	83	18.5	146	32.5	147	32.7	6	1.3
hrHPV	353	78.6	58	86.6	63	75.9	109	74.7	119	81.0	4	66.6
16	17	4.8	4	6.9	6	9.5	4	3.7	3	2.5	0	0.0
18	14	4.0	1	1.7	5	7.9	3	2.8	5	4.2	0	0.0
31	16	4.5	0	0.0	5	7.9	6	5.5	5	4.2	0	0.0
33	22	6.2	1	1.7	2	3.2	10	9.2	8	6.7	1	25.0
35	12	3.4	0	0.0	1	1.6	4	3.7	7	5.9	0	0.0
39	13	3.7	4	6.9	1	1.6	5	4.6	3	2.5	0	0.0
45	11	3.1	4	6.9	2	3.2	3	2.8	1	0.8	1	25.0
51	39	11.0	2	3.4	9	14.3	12	11.0	15	12.6	1	25.0
52	25	7.1	6	10.3	2	3.2	7	6.4	10	8.4	0	0.0
53	32	9.1	4	6.9	4	6.3	12	11.0	12	10.1	0	0.0
56	22	6.2	3	5.2	2	3.2	8	7.3	8	6.7	1	25.0
58	20	5.7	7	12.1	2	3.2	4	3.7	7	5.9	0	0.0
59	16	4.5	7	12.1	4	6.3	2	1.8	3	2.5	0	0.0
66	22	6.2	3	5.2	4	6.3	6	5.5	9	7.6	0	0.0
68	38	10.8	6	10.3	9	14.3	13	11.9	10	8.4	0	0.0
70	22	6.2	3	5.2	3	4.8	5	4.6	11	9.2	0	0.0
73	2	0.6	1	1.7	0	0.0	1	0.9	0	0.0	0	0.0
82	10	2.8	2	3.4	2	3.2	4	3.7	2	1.7	0	0.0
lrHPV	96	21.4	9	13.4	20	24.1	37	25.3	28	19.0	2	33.3
6	11	11.5	1	11.1	1	5.0	4	10.8	5	17.9	0	0.0
11	1	1.0	0	0.0	0	0.0	1	2.7	0	0.0	0	0.0
40	10	10.4	0	0.0	1	5.0	4	10.8	5	17.9	0	0.0
42	30	31.3	6	66.7	7	35.0	10	27.0	6	21.4	1	50.0
43	5	5.2	0	0.0	1	5.0	2	5.4	2	7.1	0	0.0
44/55	39	40.6	2	22.2	10	50.0	16	43.2	10	35.7	1	50.0

**Fig 2:**
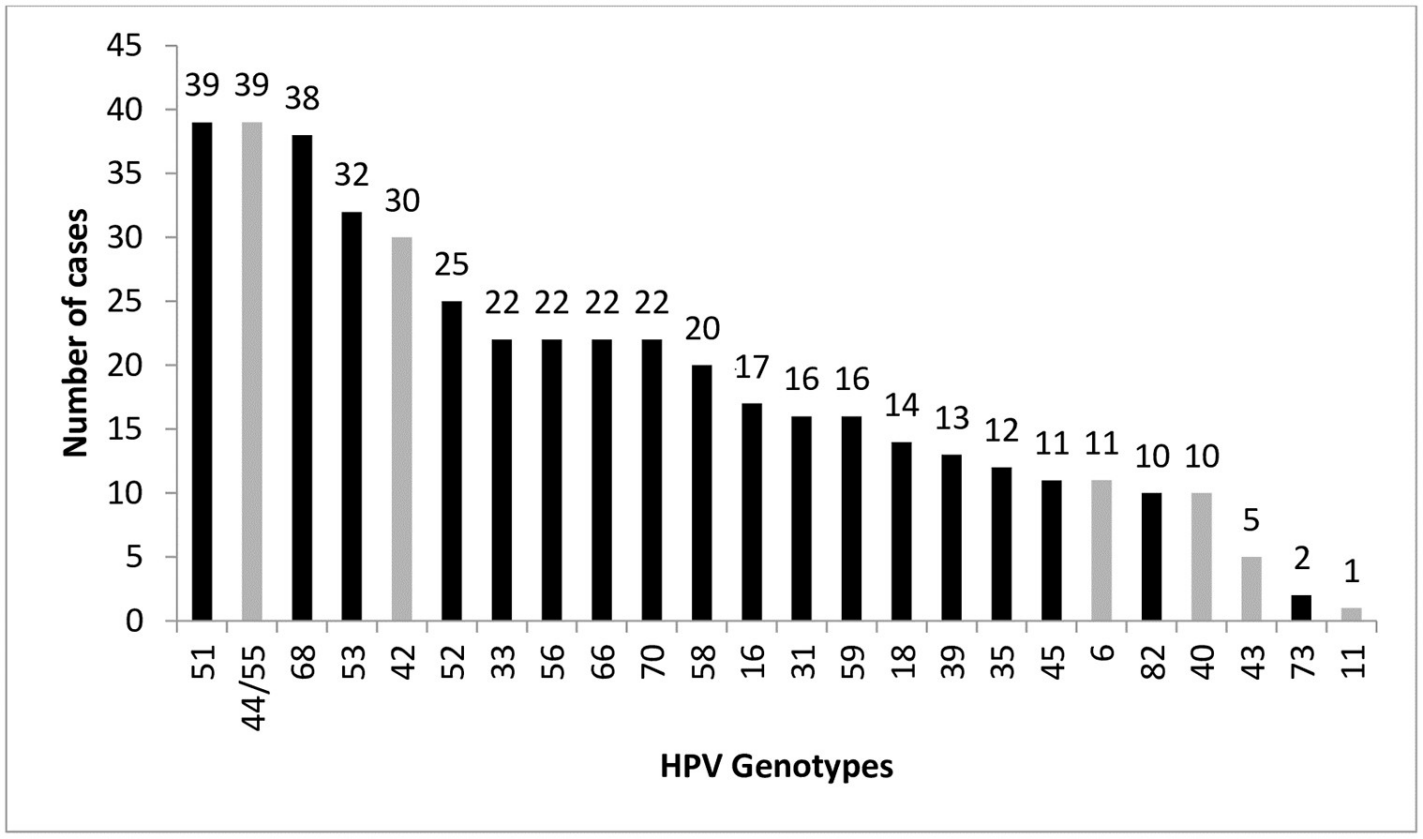
HPV genotypes in the study population in order of frequency. N = 449 cases of HPV genotypes—(hrHPV in black; lrHPV in grey).

Fig 2 shows the distribution of hrHPV genotypes in order of frequency.

**Fig 3:**
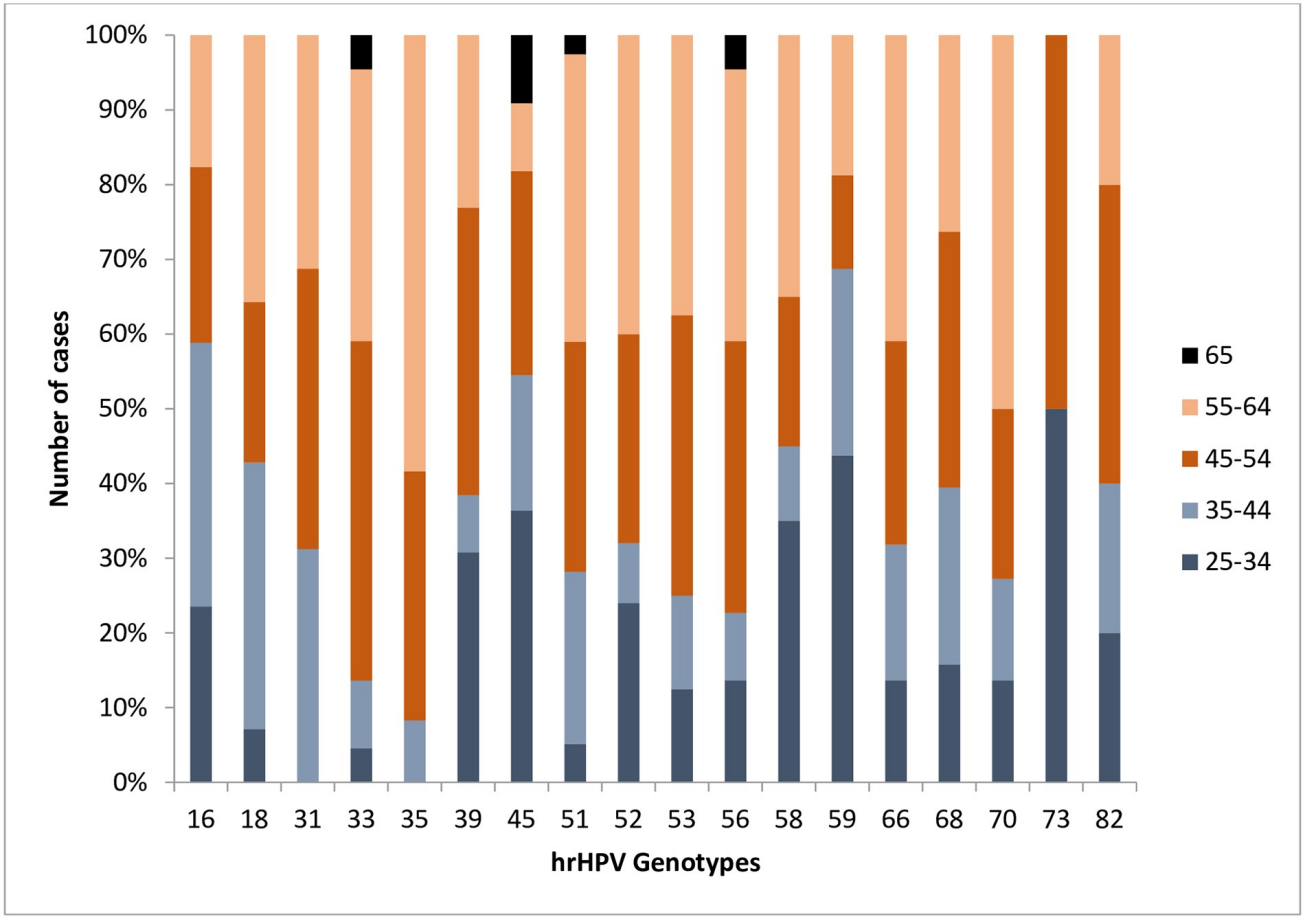
Distribution of hrHPV genotypes by age group, n = 353 women.

Fig 3 shows the hrHPV genotypes by age group. In the 25 to 44 years age group, the most frequent genotypes were HPV16, HPV45 and HPV59.

We analysed the number of co-infections with both hrHPV and lrHPV genotypes; the results are shown in Table 3.

**Table 3:** Number of coinfections by high-risk and low-risk HPV genotypes, n = 297.

	Number of hrHPVgenotypes							
	0	1	2	3	4	5	6	Total
Number of lrHPV genotypes								
n	57	164	51	16	7	1	1	297
0 lrHPV	0	147	44	11	6	0	1	209
1 lrHPV	52	17	6	3	1	1	0	80
2 lrHPV	5	0	1	2	0	0	0	8

In total, 31/297 women were co-infected with at least one hrHPV and one lrHPV genotype, corresponding to 10.4% of all women with HPV-positive results.

### Cytology

Among the 1,075 participants, 1,041 (96.8%) had normal cytology, and 225 had positive hrHPV detection (21.6%). Abnormal cytology was observed in 34 cases (3.2%), with 14 ASCUS (1.3%), 10 LSIL (0.9%), 5 HSIL (0.5%), 3 ASC-H (0.3%) and 2 AGC (0.2%). Fifteen (44.1%) were hrHPV and 4 (14.7%) lrHPV; 7 cases of hrPHV were in the 25-34-year age-group.

Among these women, the genotypes detected in the abnormal cytology samples are detailed in Table 4. We analysed the distribution of abnormal cervical lesions according to HPV genotype.

**Table 4:** Distribution of abnormal cervical lesions according to hrHPV genotype.

	hrHPV genotype														
Cytology result	16	18	33	35	39	45	51	52	53	56	58	59	66	68	82
Total abnormal cytology n = 34	4	0	1	1	2	0	1	2	3	1	2	1	4	2	3
ASC-H	1	0	0	0	0	0	0	1	0	0	0	0	0	0	1
ASC-US	0	0	0	0	0	0	0	0	2	1	0	0	1	0	0
LSIL	2	0	1	0	1	0	1	1	1	0	1	1	3	2	0
HSIL	1	0	0	1	1	0	0	0	0	0	1	0	0	0	2
Normal cytology n = 225	13	14	21	11	11	11	38	23	28	21	18	15	18	36	7

Table 5 shows the follow-up performed during the study in women with abnormal cytology, in line with the management recommendations for the study. A total of 15 women were lost to follow-up during the study and were invited to contact their treating physician for follow-up.

**Table 5:** Management in women with abnormal cytology during the study period.

Cytology	HPV-	HPV+	Colposcopy	Biopsy	LEEP
AGC	2	0	1	0	1
ASC-H	2	1	3	2	1
ASC-US	10	4	3	3	0
LSIL	1	9	7	6	0
HSIL	0	5	5	5	3
Total	15	19	19	16	5

LEEP, Loop Electrosurgical Excision Procedure.

Among the 225 women with normal cytology and positive hrHPV test, only 107 attended the study follow-up.

Of these 107 women with an initial diagnosis of hrHPV, 68 (63.5%) lost at least one hrHPV genotype (50 lost one and 18 lost 2 or more), while 13/107 (12.2%) acquired at least one new hrHPV genotype. The average time to follow-up visit for HPV 18, 35 and 52 genotypes was 3.5±1.6 years, while the average time to follow-up for all 107 women was 3.2±1.6 years.

Among the 1,075 women in the study cohort, one woman developed invasive cervical cancer, and 7 women had CIN3. The characteristics of these 8 women are presented in Table 6.

**Table 6:** Outcome of women in the study who developed cancer, as identified from the Martinique General Cancer Registry.

Type	Year of test	Year of incidence	Age at inclusion	Cytology at inclusion	Initial hrHPV			HPV at control
Cervical Cancer	2009	2013	64	Without anomaly	51	66	-	Unknown
In situ	2009	2016	63	Without anomaly	35	53	-	35 68
	2009	2011	61	Without anomaly	51	70	-	Unknown
	2011	2011	39	LSIL	16	82	-	56
	2011	2012	32	ASC-H	16	52	82	Unknown
	2011	2015	31	Without anomaly	18	70	-	none
	2011	2014	33	LSIL	39	-	-	none
	2013	2013	57	LSIL	35	-	-	Unknown

## Discussion

Few studies are available in the French West-indies on the distribution of genotypes in cervical malignancy, and this is the first study performed in Martinique in a general population-based setting. The novelty of this study is that it provides, population-based data about HPV genotype profiles in a French region of the Caribbean where there is an excess of cervical cancer.

In this study, a total of 1075 women were included, and HPV prevalence was 27.6%. The most common HPV types were HPV51 (11.0%), HPV68 (10.8%), HPV53 (9.1%) and HPV 52 (7.1%), while HPV16 and HPV 18 represented respectively 4.8% and 4.0% of hrHPV genotypes.

There is wide disparity between studies of HPV genotypes in the Caribbean, with most being carried out in samples and/or cohorts in the hospital setting. Few data are available at population level. Epidemiological studies have been performed in French Guiana [8–10] and showed epidemiological specificities in HPV genotyping. A study of 540 women of French Guiana between 2012 and 2014 showed that the most prevalent HPV genotypes were HPV 53 (3.52%), 68 (3.33%), 52 (2.59%), 31 (2.22%) and 16 (1.85%) [8].

A recent population-based study was performed in Curacao on 1075 women. Overall HPV prevalence was 19.7%. The most frequent genotypes were HPV16 (2.3%), 35 (2.1%) and 52 (1.8%); a total of 27 (2.6%) women were detected with abnormal cytology [11].

In our study, 1,041 women (96.8%) had normal cytology, and among these, 225 had positive hrHPV detection (21.6%); abnormal cytology was observed in 34 cases (3.2%).

A cross-sectional study performed in Jamaica in 2010 among 852 women reported a HPV prevalence of 34.9% (297/852). This study showed that HPV 16 or 18 was detected in 10.1% (86/852) of women. Among the 75 women with abnormal cytology results, the majority (84.0%) were HPV-positive. The HPV types were 16 (6.2%), 35 (6.0%), 58 (5.4%), 18 (4.3%),and 66 (4.2%) [12].

Another study was designed in 2014 to characterize high-risk human papillomavirus (HPV) infections in women in Saint Kitts and Nevis. High-risk HPV was detected in 102 of 404 (25.2%) in Saint Kitts and Nevis. HrHPV genotypes 52 (14.7%), 35 (13.7%), 51 (10.8%), 45 (10.8%), and 31 (10.8%) were the most common high-risk types. The majority of women in this study (94%) in Saint Kitts and Nevis who were infected with high-risk HPV had normal cytology [13].

When comparing the results of these study performed in the Caribbean, we observed that the most frequent genotypes were similar between the French territories of French Guiana and Martinique for HPV 52, 53 and 68. These two territories have also the same prevalence of 27% [8].

The 9-valent HPV vaccine (covering genotypes HPV6/11/16/18/31/33/45/52/58) was developed to expand coverage of the previous quadrivalent (HPV6/11/16/18) vaccine.

According to data on HPV genotype distribution of our study, the percentage of HPV infections which can be prevented by using a 9-valent vaccine was 47.9%. The distribution of genotypes observed in our study provides new insights into the circulating genotypes in Martinique. Among studies published heretofore from Martinique, none had provided epidemiological data at population level. Given the selection of our participants from among the clientele of a large health insurance company, it is likely that our results are not representative of the complete genotype distribution among the population of women with complementary health insurance in Martinique, due to the size of our study; this could generate a selection-bias in our study. Our results show that the distribution of genotypes in Martinique is different to that habitually observed in Europe. Of particular note is the fact that HPV 16 and 18 were not the most prevalent high-risk types in our study.

The estimated crude HPV prevalence among women with normal cytological findings worldwide was 7.2%, with the highest prevalence observed in Latin America and the Caribbean (16.1%) [14]. In our study, we found that among 1,075 women included, 34 (3.2%) had abnormal cytology, of whom 15 had a hrHPV. Among women with normal cytology, 21.6% had hrHPV detected.

We studied clearance of HPV infection among the 107 women who presented normal cytology but a hrHPV-positive test. Of these 107 women who initially had hrHPV detected, 12.2% acquired at least one new HPV genotype. The study of virus clearance is key to understanding the mechanisms mediating the development of cervical cancer after HPV infection. Our results showed the utility of implementing genotyping for the detection and monitoring of HPV infection in our population.

About 90% of HPV infections are no longer detectable after 2 years. If the HPV infection persists, it can cause cervical cancer. The persistence of a high-risk HPV infection is a necessary risk factor but other co-factors (viral, endogenous factors linked to the host or even behavioral factors) would also play a role in the genesis and development of cancer [15]. The novel findings of this study are also to analyse the persistence of the HPV in cervical cancer cases, five years after the end of the inclusion in the study.

Data from the Martinique Cancer registry showed that only one cervical cancer occurred with initial hrHPV 51 and 66 in our study.

Nonetheless, one must take into account the detection capacity of the tests, which may lead to under- or over-estimation of the prevalence of different HPV genotypes. The variability between test kits is an important point to keep in mind when comparing results between studies.

Regarding cytology, the techniques used (slides or liquid medium) may yield different results. Indeed, each commercial kit is different and sensitivity varies. Thanks to the present study, a circuit for diagnosis based on liquid medium smear test with biomolecular HPV testing was put in place, simplifying the healthcare pathway of women undergoing screening. Indeed, up to the time when this study was performed, samples were sent to mainland France for analysis.

During the period from 2015 to 2017, the standardized national coverage for 3-yearly cervical cancer screening was 58.7%. Data by geographical area reveal marked disparities, with the lowest regional coverage rates observed in Guadeloupe, French Guiana, Martinique and Mayotte (<50%) [2]. In the course of this study, we performed follow-up to assess outcomes, using data from the Martinique General Cancer Registry, which makes it possible to take into account a sufficient latency time for the development of cervical cancer. Our follow-up showed that among the original 1,075 participants, 7 developed in situ cancer and one woman had invasive cancer. The profile of the genotypes found during this follow-up showed that HPV16 and 18 were initially present, and co-infections with HPV 51, 52 and 53 were also detected.

In terms of future perspectives, analysis of HPV genotype data should make it possible in the future to cross-reference with population data such as the cancer registry database, or the social security system, to assess the healthcare pathway of women with cervical cancer. Our study does not make it possible to investigate differences that may exist in access to care or socio-economic status. Our study is useful to understand the factors explaining differences in genotype prevalence according to age and socio-economic status.

In the literature, some studies showed that there was a link between HPV infection and socioeconomic position [16,17]; in our study, there were no statistical differences according to socio-professional groups, marital status and age at diagnosis.

The next steps are to provide sufficient data to get an overview of the assessment of HPV tests in clinical practice. In fact, since this study has been performed, the French National Health Authorities developed new algorithms for cervical cancer screening including HPV test [18]; it is recommended that the HPV test replace the cytological examination as a primary screening test for cervical cancer. The results of our study could help to better identify new strategies for vaccination depending on the age of women in Martinique and to assess, in the future, the effect of the vaccination on the genotype distribution.

## Conclusion

There is wide variation between studies of HPV genotypes in the Caribbean, underlining the compelling need for multicentre comparative epidemiological studies in our region. This analysis of HPV profiles showed that the most frequent HPV genotypes were HPV51, 68, 53, namely different from those most commonly found elsewhere (HPV16, HPV18). HPV vaccination, in addition to screening by smear test, is the most effective means of preventing cancerous lesions of the cervix. Given the specificities of HPV distribution in Martinique, the implementation of a surveillance programme for HPV infection is a key public health issue for our region. Finally, these preliminary findings, from a cohort of 1075 patients, underline the necessity of accounting for heterogeneity in genotypic profiles in the surveillance and prevention of HPV-related infection in the Caribbean.
